# Adaptation of the WHO maternal near miss tool for use in sub–Saharan Africa: an International Delphi study

**DOI:** 10.1186/s12884-017-1640-x

**Published:** 2017-12-29

**Authors:** Abera K. Tura, Jelle Stekelenburg, Sicco A. Scherjon, Joost Zwart, Thomas van den Akker, Jos van Roosmalen, Sanne J. Gordijn

**Affiliations:** 1 School of Nursing and Midwifery, College of Health and Medical Sciences, Haramaya University, Harar, Ethiopia; 2 Department of Obstetrics and Gynaecology (CB20), University of Groningen, University Medical Centre Groningen, Hanzeplein 1, 9700 RB Groningen, Netherlands; 3 Department of Health Sciences, Global Health, University of Groningen, University Medical Centre Groningen, Groningen, the Netherlands; 4 Department of Obstetrics and Gynaecology, Leeuwarden Medical Centre, Leeuwarden, the Netherlands; 5 Department of Obstetrics and Gynaecology, Deventer Ziekenhuis, Deventer, the Netherlands; 6 Department of Obstetrics, Leiden University Medical Centre, Leiden, the Netherlands; 7 Athena Institute, VU University Amsterdam, Amsterdam, the Netherlands

**Keywords:** Maternal near miss, Delphi, Severe maternal morbidity, Sub–Saharan Africa, Global health

## Abstract

**Background:**

Assessments of maternal near miss (MNM) are increasingly used in addition to those of maternal mortality measures. The World Health Organization (WHO) has introduced an MNM tool in 2009, but this tool was previously found to be of limited applicability in several low–resource settings. The aim of this study was to identify adaptations to enhance applicability of the WHO MNM tool in sub–Saharan Africa.

**Methods:**

Using a Delphi consensus methodology, existing MNM tools were rated for applicability in sub-Saharan Africa over a series of three rounds. Maternal health experts from sub-Saharan Africa or with considerable knowledge of the context first rated importance of WHO MNM parameters using Likert scales, and were asked to suggest additional parameters. This was followed by two confirmation rounds. Parameters accepted by at least 70% of the panel members were accepted for use in the region.

**Results:**

Of 58 experts who participated from study onset, 47 (81%) completed all three rounds. Out of the 25 WHO MNM parameters, all 11 clinical, four out of eight laboratory, and four out of six management–based parameters were accepted, while six parameters (PaO2/FiO2 < 200 mmHg, bilirubin >100 μmol/l or >6.0 mg/dl, pH <7.1, lactate >5 μmol/l, dialysis for acute renal failure and use of continuous vasoactive drugs) were deemed to not be applicable. An additional eight parameters (uterine rupture, sepsis/severe systemic infection, eclampsia, laparotomy other than caesarean section, pulmonary edema, severe malaria, severe complications of abortions and severe pre-eclampsia with ICU admission) were suggested for inclusion into an adapted sub-Saharan African MNM tool.

**Conclusions:**

All WHO clinical criteria were accepted for use in the region. Only few of the laboratory- and management based were rated applicable. This study brought forward important suggestions for adaptations in the WHO MNM criteria to enhance its applicability in sub-Saharan Africa and possibly other low–resource settings.

## Background

In light of the global reduction in maternal mortality, assessments of severe maternal morbidity or maternal near miss (MNM) have become more common [1–3]. MNM is defined as a woman who nearly died but survived a complication that occurred during pregnancy, childbirth, or within 42 days of termination of pregnancy [3]. Different identification criteria for severe maternal morbidity have been applied in different contexts [4–6]. Application of the World Health Organization (WHO) MNM tool has become a standardized method to identify women at the severe end of the morbidity spectrum. This tool comprises three groups of criteria with clinical, laboratory and management based parameters that focus on the presence of organ dysfunction [6].

Though the WHO MNM tool has been widely used since its introduction including in sub-Saharan Africa [7–10], it also received criticism since several laboratory-based and some management-based criteria reflecting organ dysfunction turned out to be of limited relevance in resource-limited settings in sub-Saharan Africa [11, 12]. The need for more practical MNM criteria for use in low–income settings was previously noted [13] and the WHO Technical Working Group on Maternal Mortality and Morbidity classifications has indicated that an integrative module applicable to the local context for use in resource limited settings is under development [14]. Researchers have suggested possible adaptations [11, 12, 15] at the expense of inter–study comparability.

Although there is evidence that several WHO MNM parameters are not applicable to low-income settings, there is a lack of well-founded alternative parameters formulated by experts with experience in such settings [11, 12, 15]. Lack of such uniform criteria prevents robust comparison studies of MNM. The aim of this study was to come to a consensus–based adaptation of the WHO MNM tool to enhance its applicability for use in low–income settings, particularly in sub–Saharan Africa.

## Methods

We applied a three round Delphi study design. A Delphi is a structured group process in which a series of questionnaires is sent to a panel of experts who are asked to identify, rate or rank issues important to the subject under consideration [16]. It is a means of extracting opinion from a group of experts and is widely applied in medical, nursing and health services research [17–24]. We adopted a quasi–anonymous Delphi, in which each panel member was aware of the other participating members, but responses were kept anonymous and presented on a group level. In our opinion, it was important for members to be informed about the composition of the group in order to assign appropriate value to the panel. In every subsequent round, group responses were reported and instructions for completion of that round were provided. Questionnaire development, pre-testing, analysis and coordination were conducted by AKT and SJG. A Delphi steering committee consisting of all authors (AKT, SAS, JS, JZ, JvR, TvdA, and SJG) was established to coordinate the Delphi process, analyse comments and determine a priori criteria for consensus and termination of the study.

## Selection and recruitment panel of experts

Expert panel members were selected if they authored an article on maternal near miss in sub-Saharan Africa [25] or if they were suggested by already selected authors. All experts were approached through email by introducing purpose of the study, its design, and a request to participate in the expert panel. We aimed to obtain wide coverage of experts with experience throughout sub-Saharan Africa and therefore no further selection was done after agreement for participation. After agreeing to participate, they were included in the first round and invited for subsequent rounds only if they completed the former round.

## The Delphi procedure

Questionnaires were developed using LimeSurvey version 2.05+ (www.limesurvey.org) and sent with a unique, token secured link to participants using email. Three rounds of sequential online Delphi surveys were administered. Each round of Delphi was conducted over a three-week period with 2 to 3 months between rounds for analysis, questionnaire refinement and pilot testing. Email reminders were sent for non-respondents approximately after 10 days followed by an additional two reminders. Members of the steering committee (JS, JZ, TvdA and JvR) did take part as experts within the survey.

We used the 2009 MNM tool that consisted of 25 parameters, as well as an additional 12 parameters from the literature [4–6, 11, 26] in round 1 (Table 1). For each parameter, participants were asked to indicate their level of agreement on a 5-point Likert scale (ranging from 1 = least important to 5 = most important) as to whether that parameter would be important for identification of MNM in sub-Saharan Africa. A free-text field was also provided, inviting panel members to suggest additional parameters to be considered as criteria for MNM. Sociodemographic characteristics, professional background, country of work and experience of participants in sub-Saharan Africa were collected in round 1which was conducted in October 2015.

**Table 1:** List of parameters presented for evaluation and suggested in the study

Original parameters	Suggested parameters (=14)
WHO Parameters (=25)	
1. Acute cyanosis 2. Gasping 3. Respiratory rate > 40 or <6/min 4. Shock 5. Oliguria non responsive to fluids or diuretics 6. Failure to form clots 7. Loss of consciousness lasting more than 12 h 8. Cardiac arrest 9. Stroke 10. Uncontrollable fit/total paralysis 11. Jaundice in the presence of pre-eclampsia 12. Oxygen saturation < 90% for >60 min 13. PaO2/FiO2 < 200 mmHg 14. Creatinine >300 μmol/l or >3.5 mg/dl 15. Bilirubin >100 μmol/l or >6.0 mg/dl 16. pH <7.1 17. Lactate >5 mEq/ml 18. Acute thrombocytopenia (<50,000 platelets/ml) 19. Loss of consciousness and ketoacids in urine 20. Use of continuous vasoactive drugs 21. Hysterectomy following infection or haemorrhage 22. Massive transfusion of blood or red cells (≥5 units) 23. Intubation and ventilation for >60 min not related to anaesthesia 24. Dialysis for acute renal failure 25. Cardio-pulmonary resuscitation Parameters from the literature (=12) 26. Uterine rupture 27. Sepsis or severe systemic infection 28. Eclampsia 29. Laparotomy other than CS 30. Pulmonary edema 31. Admission to the ICU 32. Diabetic Keto Acidosis 33. Severe malaria 34. Obstructed labour 35. Severe anaemia 36. Severe HIV related illnesses 37. Uterine artery embolization	1. Severe abortion complications 2. Failed tracheal intubation requiring anaesthetic reversal 3. Maternal indication to terminate pregnancy 4. Kussmaul respiration 5. Severe dehydration 6. Confusion 7. Ketotic breath 8. Acute kidney injury 9. Uterine tamponade 10. Ligation of internal iliac vessels 11. Severe hypotension (SBP < 90 mmHg lasting >60 min) 12. Pre-eclampsia with the presence of oliguria or respiratory disorder 13. Severe PPH (>1000 ml of blood) within 24 h of delivery 14. Severe pre-eclampsia with ICU admission

*CS* Caesarean Section, *ICU* Intensive Care Unit, *HIV* Human Immunodeficiency Virus, *SBP* Systolic Blood Pressure, *PPH* Postpartum haemorrhage

In round 2, which was conducted in December 2015, we grouped parameters from round 1 in to *accepted, maybe accepted* and *rejected* based on their median score of 5, 4, and ≤3 respectively. A fourth group of *suggested* parameters was also constructed from parameters suggested for inclusion by participants in round 1. These were presented to the panel for verification of the groups using a yes-no question. Consensus for inclusion of an item for use in an adapted ‘sub-Saharan MNM tool’ was defined a priori as at least 70% agreement. Level of agreement of at least 70% was used in several Delphi studies as level consensus [17, 18]. A parameter that failed to receive at least 60% would be excluded. Parameters with rates of agreement between 60% and 70% were brought back for voting again in round 3. We asked the panel to indicate for every accepted parameter which definitions or cut off values should be considered by providing drafts of definitions and suggested cut-off points.

In round 3, conducted in May 2016, we included all parameters for which consensus was not reached for final voting. A list of parameters which reached consensus for inclusion or exclusion were also presented for their information only. The cut-off point for inclusion remained 70%. All responses were analysed using SPSS version 23.

## Results

### Participants

Of 102 experts invited for participation, 58 (56.9%) agreed to participate. Fifty two out of 58 (89.7%) completed round 1, 50/52 (96.2%) round 2, and 47/49 (95.9%) all three rounds. One participant opted out from participation after round 2 due to internet connection problems. Twenty–two countries were represented by the expert panel, the majority from sub–Sahara Africa: one each from Belgium, Benin, Ghana, Kenya, Malawi, Mozambique, Norway, Rwanda, Sudan, Switzerland and United States of America; two from Italy; three each from Burkina Faso, Sweden, Uganda and United Kingdom; four each from Brazil, Ethiopia, Nigeria, South Africa, and Tanzania; and seven from the Netherlands where the study was initiated.

Most experts were male (*n* = 40; 76.9%), obstetricians (n = 40; 76.9%), and had a PhD degree (*n* = 28; 53.8%). The majority (44; 84.6%) had more than 5 years of experience within their current position and 34 (65.4%) had five or more years of work experience in sub-Saharan African settings. Four were members of the WHO working group on Maternal Mortality and Morbidity classifications who developed the WHO 2009 MNM tool [3, 6].

### First round

**Fig. 1:**
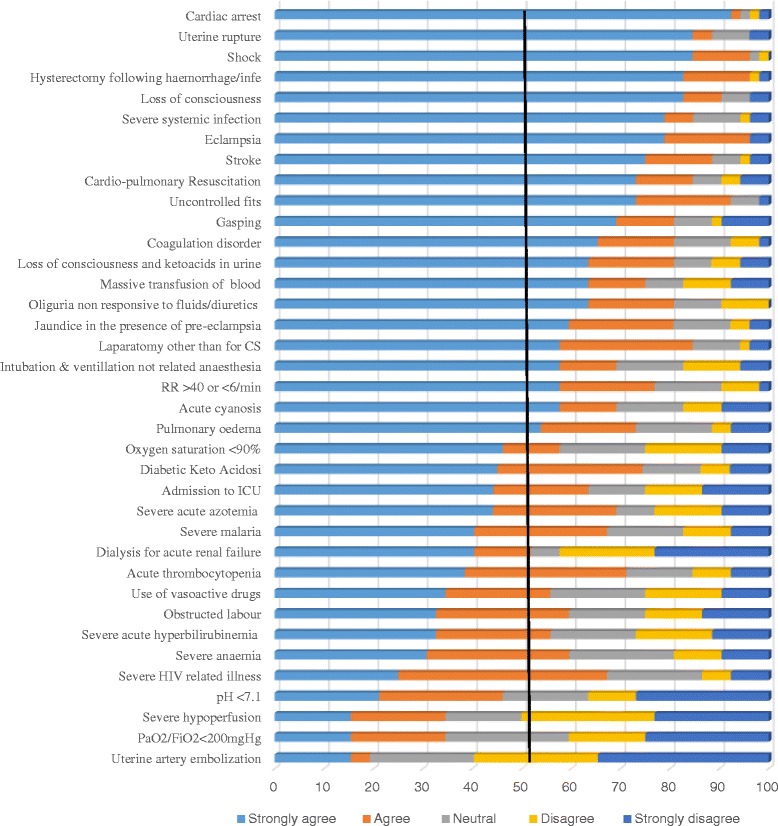
Level of agreement for using selected MNM parameters in sub–Saharan Africa (round 1)

Of the 37 parameters presented for evaluation, 21(56.8%) were rated as ‘very important’ (16 of 25 WHO parameters and 5 of 12 literature-based parameters). Twelve (6 of 25 WHO and 6 of 12 literature-based parameters) were rated as ‘may be important’ while 4 parameters (3 of 25 WHO, 1 of 12 literature-based) were rated as ‘unimportant’. Cardiac arrest, shock, loss of consciousness lasting more than 12 h and haemorrhage or infection leading to hysterectomy achieved greatest consensus with >80% of experts rating these as ‘very important’. Contrastingly, PaO2/FiO2 < 200 mmHg, lactate >5 mEq/ml, pH <7.1 and uterine artery embolization received very low rates of agreement (<22%) for use in the region. Specific levels of agreement for each parameter in round 1 are shown in Fig. 1. Several additional parameters (*n* = 31) were suggested for consideration by 19 panel members. All suggested parameters were collected by two authors (AKT and SJG) and presented to the steering committee for discussion. Parameters were discussed for their importance and were combined in case of overlap. Following the discussion among the Delphi steering committee, 14 parameters were summarized and reported back to the experts as ‘*suggested* parameters’ in round 2.

### Second and third rounds

In round 2, consensus was reached to include 26 parameters (18 from WHO, 6 from the literature and two from suggested parameters. For accepted parameters, consensus ranged from 100% for shock and cardiac arrest to 71.7% for severe pre–eclampsia with ICU admission. In this round, 15 other parameters were voted to be excluded. This includes four parameters from WHO (PaO2/FiO2 < 200 mmHg, pH <7.1, bilirubin >100 μmol/l or >6.0 mg/dl, and lactate >5 mEq/ml), two from the literature based (obstructed labour and interventional radiology), and nine from the suggested parameters (Tables 2 and 3). Among rejected parameters consensus rates for inclusion of the MNM tool ranged from 6.5% for uterine artery embolization to 54.3% for obstructed labour. For 10 parameters, consensus was not reached for inclusion or exclusion. There was also no consensus on cut–off points for number of units of blood transfused to the mother as a proxy for major obstetric haemorrhage. The 10 parameters and suggested cut-off points for blood transfusion were therefore reported again in round 3 for rating.

**Table 2:** Rate of agreement on inclusion of selected MNM parameters for use in sub-Saharan Africa

Parameter	Round 1 (*n* = 52)							Round 2(*n* = 50)		Round 3 (*n* = 47)	
	Strongly Agree	Agree	Neutral	Disagree	Strongly disagree	Median	Decision	%	Decision	%	Decision
1. Cardiac arrest	48	1	1	1	1	5	Accepted	100	Accepted	–	–
2. Shock	44	6	1	1	0	5	Accepted	100	Accepted	–	–
3. Loss of consciousness lasting >12 h	43	4	3	0	2	5	Accepted	98	Accepted	–	–
4. Hysterectomy for haemorrhage or infection	43	7	0	1	1	5	Accepted	98	Accepted	–	–
5. Stroke	39	7	3	1	2	5	Accepted	92	Accepted	–	–
6. Uncontrolled fits/total paralysis	38	10	3	0	1	5	Accepted	96	Accepted	–	–
7. Cardiopulmonary resuscitation	38	6	3	2	3	5	Accepted	98	Accepted	–	–
8. Gasping	36	6	4	1	5	5	Accepted	92	Accepted	–	–
9. Failure to form clots/coagulation disorder	34	8	6	3	1	5	Accepted	96	Accepted	–	–
10. Oliguria non-responsive to fluids or diuretics	33	9	5	5	0	5	Accepted	92	Accepted	–	–
11. Transfusion of blood	33	6	4	5	4	5	Accepted	90	Accepted	–	–
12. Loss of consciousness & ketoacidosis in urine	33	9	4	3	3	5	Accepted	90	Accepted	–	–
13. Jaundice in the presence of pre-eclampsia	31	11	6	2	2	5	Accepted	84	Accepted	–	–
14. Acute cyanosis	30	6	7	4	5	5	Accepted	92	Accepted	–	–
15. Respiration rate > 40 or <6/min	30	10	7	4	1	5	Accepted	94	Accepted	–	–
16. Intubation & ventilation not related to anaesthesia	30	6	7	6	3	5	Accepted	92	Accepted	–	–
17. Oxygen saturation < 90% for ≥60 min	24	6	9	8	5	4	May be accepted	80·4	Accepted	–	–
18. Creatinine ≥300 μmol/l or ≥3.5 mg/dl	23	13	4	7	5	4	May be accepted	73·9	Accepted	–	–
19. Dialysis for acute renal failure	21	6	3	10	12	4	May be accepted	67·4	May be accepted	66	Rejected
20. Acute thrombocytopenia <50,000/ml)	20	17	7	4	4	4	May be accepted	69·6	Maybe accepted	72·3	Accepted
21. Use of continuous vasoactive drugs	18	11	10	8	5	4	May be accepted	67·4	May be accepted	61·7	Rejected
22. Bilirubin >100 μmol/l or >6.0 mg/dl	17	12	9	8	6	4	May be accepted	52·2	Rejected	–	–
23. pH <7.1	11	13	9	5	14	3	Rejected	21·7	Rejected	–	–
24. PaO2/Fi2 < 200 mmHg	8	10	13	8	13	3	Rejected	18	Rejected	–	–
25. Lactate >5 mEq/ml	8	10	8	14	12	2	Rejected	17·4	Rejected	–	–
26. Uterine rupture^a^	44	2	4	0	2	5	Accepted	94	Accepted	–	–
30. Sepsis or severe systemic infection^a^	41	3	5	1	2	5	Accepted	90	Accepted	–	–
34. Eclampsia^a^	41	9	0	0	2	5	Accepted	88	Accepted	–	–
28. Laparotomy other than CS^a^	30	14	5	1	2	5	Accepted	78	Accepted	–	–
27. Pulmonary edema^a^	28	10	8	2	4	5	Accepted	78	Accepted	–	–
29. Admission to the ICU^a^	23	10	6	6	7	4	May be accepted	63	May be accepted	59·6	Rejected
36. Diabetic Keto Acidosis^a^	23	15	6	3	4	4	May be accepted	65·2	May be accepted	61·7	Rejected
33. Severe malaria^a^	21	14	8	5	4	4	May be accepted	73·9	Accepted	–	–
31. Obstructed labour^a^	17	14	8	6	7	4	May be accepted	54·3	Rejected	–	–
32. Severe anaemia^a^	16	15	11	5	5	4	May be accepted	69·6	May be accepted	61·7	Rejected
35. Severe HIV related illnesses^a^	13	22	10	3	4	4	May be accepted	69·6	May be accepted	57·4	Rejected
37. Interventional radiology^a^	8	2	11	13	18	2	Rejected	6·5	Rejected	–	–

*CS* Caesarean Section, *ICU* Intensive Care Unit, May be accepted

^a^ parameters from literature

**Table 3:** Suggested parameters and respective decisions in round 2 and round 3

Parameter	Round 2 n(% agree)	Round 3 n(% agree)	Final Decision
1. Severe abortion complications	36(78·3)	–	Accepted
2. Failed tracheal intubation requiring anaesthetic reversal	17(37·0)	–	Rejected
3. Maternal indication to terminate pregnancy	13(28·3)	–	Rejected
4. Kussmaul respiration	18(39·1)	–	Rejected
5. Severe dehydration	14(30·4)	–	Rejected
6. Confusion	18(39·1)	–	Rejected
7. Ketotic breath	17(37·0)	–	Rejected
8. Acute kidney injury	22(47·8)	–	Rejected
9. Uterine tamponade	20(43·5)	–	Rejected
10. Ligation of internal iliac vessels	22(47·8)	–	Rejected
11. Severe hypotension (systolic BP <90 mmHg lasting >60 min)	28(60·9)	21(44·7)	Rejected
12. Pre-eclampsia with the presence of oliguria or respiratory disorder	32(69·6)	30(63·8)	Rejected
13. Severe PPH (loss of more than 1000 ml of blood) within 24 h of delivery	29(63·0)	31(66.0)	Rejected
14. Severe pre-eclampsia with intensive care unit admission	33(71·7)	–	Accepted

Note: these parameters were suggested in round 1; so rating was done in round 2 and 3 only. *BP* blood pressure, *PPH* postpartum haemorrhage

In round 3, only one parameter (acute thrombocytopenia, platelets <50,000/ml) was accepted with a level of agreement of 72.3%. Nine parameters including dialysis for acute renal failure and use of continuous vasoactive drugs from the WHO parameters were excluded. Other literature based parameters: admission to the intensive care unit, diabetic ketoacidosis, severe anaemia and severe HIV related illness were also rejected. Detailed level of agreement, the Delphi rounds and corresponding decisions is shown in Tables 2 and 3.

### Adapted sub-Saharan Africa MNM tool

At the end of this Delphi exercise, 27 MNM parameters were accepted for use in sub-Saharan Africa. This includes 19 parameters from WHO 2009 parameters (11 out of 11 clinical, four out of eight laboratory-based, and four out of six management-based criteria). Additionally, eight parameters were accepted for MNM criteria in the region (six out of 12 literature based criteria and two from *suggested* parameters). These include seven clinical (eclampsia, pulmonary edema, ruptured uterus, severe complications of abortion, severe malaria sepsis/severe systemic infection and severe pre-eclampsia with ICU admission) and one management-based (laparotomy other than caesarean section) parameters. Consensus was reached on working definitions and cut-off values for the majority of the newly added parameters while the existing WHO definition was taken up for original parameters. Consensus was not reached for the number of units of blood to constitute MNM due to haemorrhage. Eighteen (38.3%) experts suggested basing the need for blood transfusion, followed by 16(34%) and 14(29.8%) who opted using five and two units of blood transfusion respectively. The final set of ‘sub-Saharan Africa MNM Tool’ parameters with their respective definitions is shown in Table 4.

**Table 4:** Adapted sub-Saharan Africa MNM tool

WHO maternal near miss criteria	sub-Saharan Africa maternal near miss criteria
Clinical criteria	
Acute cyanosis	Acute cyanosis ^a^
Gasping	Gasping ^b^
Respiratory rate > 40 or <6/min	Respiratory rate > 40 or <6/min
Shock	Shock ^c^
Oliguria non responsive to fluids or diuretics	Oliguria non responsive to fluids or diuretics ^d^
Failure to form clots	Failure to form clots ^e^
Loss of consciousness lasting more than 12 h	Loss of consciousness lasting more than 12 h ^f^
Cardiac arrest	Cardiac arrest
Stroke	Stroke ^g^
Uncontrollable fit/total paralysis	Uncontrollable fit/total paralysis ^h^
Jaundice in the presence of pre-eclampsia	Jaundice in the presence of pre-eclampsia ^i^
	Eclampsia ^j^
	Uterine rupture ^k^
	Sepsis or severe systemic infection ^l^
	Pulmonary edema ^m^
	Severe abortion complications ^n^
	Severe malaria ^o^
	Severe pre-eclampsia with ICU admission
Laboratory based criteria	
Oxygen saturation < 90% for > 60 min	Oxygen saturation < 90% for > 60 min
PaO2/FiO2 < 200 mmHg	
Creatinine ≥ 300 μmol/l or ≥ 3.5 mg/dl	Creatinine ≥ 300 μmol/l or ≥ 3.5 mg/dL
Bilirubin > 100 μmol/l or > 6.0 mg/dl	
pH <7.1	
Lactate > 5 mEq/ml	
Acute thrombocytopenia (<50,000 platelets/ml)	Acute thrombocytopenia (<50,000 platelets/ml)
Loss of consciousness and ketoacids in urine	Loss of consciousness and ketoacids in urine
Management based criteria	
Use of continuous vasoactive drugs	
Hysterectomy following infection or haemorrhage	Hysterectomy following infection or haemorrhage
Transfusion of ≥ 5 units of blood	Transfusion of ≥ 2 units of red blood cells
Intubation and ventilation for ≥ 60 min not related to anaesthesia	Intubation and ventilation for ≥ 60 min not related to anaesthesia
Dialysis for acute renal failure	
Cardio-pulmonary resuscitation	Cardio-pulmonary resuscitation
	Laparotomy other than caesarean section

^a^Acute cyanosis is blue or purple colouration of the skin or mucous membranes due to low oxygen saturation

^b^Gasping is a terminal respiratory pattern and the breath is convulsively and audibly caught

^c^Shock is persistent severe hypotension, defined as a systolic BP <90 mmHg for ≥ 60 min with a pulse rate at least 120 despite aggressive fluid replacement (> 2 L)

^d^Oliguria is urinary output <30 ml/h for 4 h or <400 ml/24 h

^e^Failure to form clots can be assessed by the bedside clotting test or absence of clotting from the IV site after 7–10 min

^f^Loss of consciousness lasting > 12 h is a profound alteration of mental state that involves complete or near-complete lack of responsiveness to external stimuli. It is defined as a Glasgow Coma Scale <10 (moderate or severe coma)

^g^Stroke is neurological deficit of cerebrovascular cause that persists beyond 24 h or is interrupted by death within 24 h

^h^Uncontrolled fits/total paralysis is refractory, persistent convulsions or status epilepticus

^I^Pre-eclampsia is defined as the presence of hypertension associated with proteinuria. Hypertension is defined as a BP of at least 140/90 mmHg on at least two occasions and at least 4-6 h apart after the 20th week of gestation in women known to be normotensive beforehand. Proteinuria is defined as excretion of 300 mg or more of protein every 24 h. If 24-h urine samples are not available, proteinuria is defined as a protein concentration of 300 mg/l or more (≥ 1 on dipstick) in at least two random urine samples taken at least 4–6 h apart

^j^Eclampsia is diastolic BP ≥ 90 mmHg or proteinuria +3 and convulsion or coma

^k^Uterine rupture is complete rupture of uterus during labour and/or confirmed later by laparotomy

^l^Sepsis or severe systemic infection is defined as a clinical sign of infection and 3 of the following: temp > 38 °C or <36 °C, respiration rate >  20/min, pulse rate >  90/min, WBC > 12,000

^m^Pulmonary edema is accumulation of fluids in the air spaces and parenchyma of the lungs

^n^Severe abortion complications is defined as septic incomplete abortion, or complicated gestational trophoblastic disease with anaemia

^o^Severe malaria is defined as major signs of organ dysfunction and/or high level parasitemia or cerebral malaria

## Discussion

To the best of our knowledge, this is the first study bringing together the opinions of a large group of experts concerning the construction of a feasible set of MNM criteria for use in low resource sub–Saharan African settings. The majority of the WHO MNM tool parameters were rated feasible for use in sub-Saharan Africa. On the other hand, our Delphi experts rated several laboratory and management-based parameters not to be feasible. On several clinical criteria, initially not included in the WHO MNM tool, consensus was reached and these are suggested to be added. Hence, we provided a framework of an adapted MNM tool with 27 parameters for use in sub-Saharan Africa.

We followed the structure of the existing WHO MNM tool to suggest inclusion or exclusion of parameters [6]. The adapted MNM parameters have the potential to serve as uniform adaptations and enable inter-study comparisons in the future. We followed recommended Delphi practices: reproducible participant criteria, a priori defined level of consensus for inclusion and exclusion of parameters, and a planned number of rounds [27, 28].

A majority of the WHO 2009 MNM parameters in general and the clinical criteria in particular were found to be acceptable criteria for use in sub-Saharan Africa. These results are in line with adaptations suggested in Rwanda [15], Tanzania [11], and Malawi [12].

Our results also favour inclusion of several clinical criteria, which were not part of the 2009 WHO MNM parameters. Such parameters were previously part of the recommendations by researchers who tried application of the tool in low-income settings [11, 12, 15]. Out of these eclampsia, ruptured uterus, and sepsis/severe systemic infection are classified as ‘potentially life threatening complications’ of pregnancy by WHO [3, 6]. Inclusion of these potentially life threatening complications as additional parameters for MNM is supported by a recent study in maternity units in Latin America indicating that the likelihood of developing severe maternal outcomes (MNM & MD) was high among cases with many these potentially life threatening complications [29]. A study from Malawi and other African countries that reported that these ‘potentially life threatening complications’ have high case fatality rates [30, 31].

In this study, only few of the laboratory- and management-based parameters were accepted into the sub-African MNM Tool. The use of laboratory, and management-based parameters in low-income settings is more problematic due to lack of laboratory facilities and qualified health staffs in many settings [11, 12, 15].

Consensus was not reached regarding the number of units of blood for transfusion as a criterion for major obstetric haemorrhage. In our opinion it is slightly alarming that more than one in three (34%) panel members suggested that postpartum haemorrhage does not become life threatening in sub-Saharan Africa until five units of blood are administered, given the serious lack of blood for transfusion. In many district hospitals, it is very rare to have five units of blood available for transfusion. This renders a definition of MNM based on a number of transfusions highly problematic [32]. Although we tried to overcome this by providing an option based on the need for blood transfusion had it been available, no consensus was achieved. We strongly feel that a criterion of five units would underestimate the magnitude of MNM in the region. Hence, from a pragmatic point of view, we suggest the use of at least two units of blood as a cut–off, taking into account lack of blood for transfusion in the region. A cut-off of two units was found effective previously [11, 12].

This study involved participation of international maternal health experts, including experts involved in the development of the 2009 WHO MNM tool, from 22 countries with considerable expertise in sub-Saharan Africa. The views of included experts expressed here, however, may differ from those who declined or did not participate and outcomes do not necessarily represent the views of individual participants. Most of our expert panel members were male obstetricians, which is a reflection of the experts working in sub-Saharan Africa. We do not expect that another composition of the experts based on gender or professional background would influence the results. The use of the online system prevented bias towards strong voice [23, 28]. Participation was entirely voluntary, and all panel members were given the opportunity to withdraw from the survey at any stage.

Findings were limited by lack of arranging consultative meetings for parameters which were still undetermined in round 3. Consultative meetings were found to be effective in solving issues related to equivocal findings in other studies [17]. Arranging consultative meeting or video-conference was impossible due to financial and technological restrictions.

## Conclusion

A majority of WHO MNM parameters were rated to be feasible for use in sub-Saharan Africa. Although the WHO MNM tool aims to enable comparisons between settings, and context-based adjustments of the tool may hamper inter–study comparability [6, 33], we feel that this aim becomes irrelevant unless the WHO tool is adjusted for use in low–resource settings where most MNM occurs [13]. In order to increase comparability of future studies, we recommend that researchers apply these adapted criteria for case selection. There is a need to assess specificity, sensitivity, and predictive value of the adapted tool compared to the WHO tool for use in sub-Saharan Africa or other low income settings [12, 34]. We hope that our results may be taken into account by WHO in their valued advocacy for studies into MNM, including comparisons. In addition, our results may feed into the Core Outcomes in Women’s and Newborn Health Initiative (CROWN) [35] and our adapted tool may promote standardized outcome reporting in low-resource settings.

## Abbreviations

CS: Caesarean section
ICU: Intensive care unit
MNM: Maternal near miss
PPH: Postpartum hemorrhage
WHO: World Health Organization
